# Intramuscular fat in ambulant young adults with bilateral spastic cerebral palsy

**DOI:** 10.1186/1471-2474-15-236

**Published:** 2014-07-12

**Authors:** Jonathan J Noble, Geoffrey D Charles-Edwards, Stephen F Keevil, Andrew P Lewis, Martin Gough, Adam P Shortland

**Affiliations:** 1 Division of Imaging Sciences and Biomedical Engineering, King’s College London, The Rayne Institute, 4th Floor, Lambeth Wing, St Thomas’ Hospital, London SE1 7EH, United Kingdom; 2 Department of Medical Physics, Guy’s and St Thomas’ NHS Foundation Trust, The Rayne Institute, 4th Floor, Lambeth Wing, St Thomas’ Hospital, London SE1 7EH, United Kingdom; 3 One Small Step Gait Laboratory, Guy’s and St Thomas’ NHS Foundation Trust, Guy’s Hospital, London SE1 9RT, United Kingdom

**Keywords:** Cerebral palsy, Intramuscular fat, Magnetic resonance imaging, Dixon imaging

## Abstract

**Background:**

It is known that individuals with bilateral spastic cerebral palsy (BSCP) have small and weak muscles. However, no studies to date have investigated intramuscular fat infiltration in this group. The objective of this study is to determine whether adults with BSCP have greater adiposity in and around their skeletal muscles than their typically developing (TD) peers as this may have significant functional and cardio-metabolic implications for this patient group.

**Methods:**

10 young adults with BSCP (7 male, mean age 22.5 years, Gross Motor Function Classification System (GMFCS) levels I-III), and 10 TD young adults (6 male, mean age 22.8 years) took part in this study. 11 cm sections of the left leg of all subjects were imaged using multi-echo gradient echo chemical shift imaging (mDixon). Percentage intermuscular fat (IMAT), intramuscular fat (IntraMF) and a subcutaneous fat to muscle volume ratio (SF/M) were calculated.

**Results:**

IntraMF was higher with BSCP for all muscles (p = 0.001-0.013) and was significantly different between GMFCS levels (p < 0.001), with GMFCS level III having the highest IntraMF content. IMAT was also higher with BSCP p < 0.001). No significant difference was observed in SF/M between groups.

**Conclusion:**

Young adults with BSCP have increased intermuscular and intramuscular fat compared to their TD peers. The relationship between these findings and potential cardio-metabolic and functional sequelae are yet to be investigated.

## Background

The mechanical potential of muscle depends on gross morphology (cross sectional area, fibre length [1]), muscle fibre type, and muscle composition (fraction of intramuscular fat and connective tissue). Anatomical magnetic resonance imaging (MRI) and ultrasound imaging have demonstrated that the skeletal muscles of the lower limbs of individuals with bilateral spastic cerebral palsy (BSCP) are reduced in size by up to 50% compared to the muscles of their typically developing (TD) peers [2-4]. However, studies of microstructure and composition have been limited to a small number of biopsy studies [5,6]. In the lower limb, these have demonstrated increases in the connective tissue fraction, reduced muscle fibre diameter, and altered muscle fascicle stiffness [5,6].

Decreased physical activity is associated with increased intermuscular fat [7-10]. Due to the typically sedentary behaviour observed in this group [11,12], one aspect of muscle composition that may be altered in BSCP is the level of intra- and intermuscular fat. Fatty infiltration into muscle results in a reduced proportion of contractile tissue per unit muscle volume. Intramuscular fat also may secrete inflammatory cytokines that can reduce the myofibrillar force even in the absence of muscle atrophy [13]. If present in this group, raised intramuscular fat may have important consequences for physical performance, and implications for exercise regimes employed in their physical management [14].

Heightened levels of inter- and intra- muscular fat have been shown to be associated with cardiovascular risk [15,16]. Adults with cerebral palsy may have a 2–3 times greater risk of dying from ischemic heart disease than their typically developing peers [17]. However, there are few studies in the literature that have attempted to document body or muscle composition in this group. Since individuals with cerebral palsy have reduced muscle mass, even those with body mass index (BMI) in the normal range may have relatively increased levels of adipose tissue. Previous studies in children with cerebral palsy have suggested raised levels of fat [18,19]. In an MRI study of the lower limbs in children with quadriplegic cerebral palsy (Gross Motor Function Classification System (GMFCS) levels III-V), Johnson *et al.*[18] found increased levels of intermuscular and subcutaneous fat [18]. To date, there have been no studies of intramuscular fat in adults or children with BSCP even though this non-invasively quantified parameter appears to be one of those most associated with cardio-metabolic disease [20].

T_1_-weighted image segmentation is primarily used to quantify IMAT, with IMAT defined as the MRI-visible fat within the muscles (intramuscular fat) and between the muscles beneath the fascia (intermuscular fat). Although this method is effective for assessing intermuscular and subcutaneous fat volumes, it is not an accurate method for measuring fat distribution within individual muscles. Chemical shift magnetic resonance imaging-based water/fat separation techniques based on that first proposed by Dixon [21] have been developed which utilise the chemical shift difference between fat and water to enable reconstruction of separate water and fat images. These techniques utilise the predictable difference in phase evolution between water and fat signals due to their chemical shift difference to enable the calculation of separate water and fat images, permitting the calculation of the fat fraction. The fat fraction is the signal intensity attributable to fat, normalised by the total signal from all mobile proton species. These techniques have been widely used in studies quantifying the degree of liver fat in hepatic steatosis (for review see Reeder [22]): although, to date, Dixon imaging has only been used in a small number of clinical intramuscular fat quantification studies [16,23,24].

The aim of this study is to investigate the intermuscular fat (IMAT), subcutaneous fat (SF) and intramuscular fat (IntraMF) content in five major muscles of the leg (medial and lateral gastrocnemius, soleus, tibialis posterior and tibialis anterior) in 10 subjects with BSCP and 10 TD subjects using multi-echo gradient echo chemical shift imaging (mDixon). We hypothesised that the subjects with BSCP would exhibit increased fat content compared to their TD peers.

## Methods

Ethical approval for this study was granted by Hampstead Research Ethics Committee London (09/H0720/120). All subjects gave informed consent before they participated.

### Subjects

Individuals aged 16 – 30 years, with a diagnosis of BSCP, Gross Motor Function Classification System (GMFCS) levels I-III, who met the safety requirements of MRI were included in this study. Patients who had undergone surgery, serial casting or botulinum toxin injections to the lower limbs within the previous year were excluded from the study. This was a convenience sample of individuals attending our hospital department, with consecutive patients that met the inclusion criteria invited to participate in the study.

10 adults with a diagnosis of BSCP (7 male, 3 female, mean age 22.5 years, range 18–27, GMFCS levels I-III from clinics in our university hospital and a convenience sample of 10 TD young adults (6 male, 4 female, mean age 22.8 years, range 18–27) recruited from individuals known to the research team participated in this study. The BSCP subjects had undergone a range of previous interventions (see Table 1) but none had undergone surgery, serial casting or botulinum toxin injections to the lower limbs within the previous year. All TD subjects had no prior significant musculoskeletal trauma or disorders.

**Table 1:** Previous left leg interventions in the group of adults with BSCP

**BSCP subject**	**Intervention**
1	None
2	Gastrocnemius lengthening
3	Gastrocnemius lengthening
4	None
5	Gastrocnemius lengthening
6	Gastrocnemius lengthening
7	Gastrocnemius lengthening
8	None
9	Gastrocnemius and Achilles tendon lengthening
10	None

### Data collection

MR data were acquired on a 3.0 T Achieva system (Philips Healthcare, Best, The Netherlands) running software version 2.6.3, using an 8-channel receive-only phased array knee coil. One subject had a 30° knee fixed flexion deformity; instead a 32-channel cardiac coil was used. An 11 cm section of the left leg was scanned, centred at the largest circumference of the calf with the subject prone. Four point gradient echo mDixon images were acquired with TE/TR = 2.3/7.1 ms, 35° flip angle, 2.0 × 2.0 mm in-plane resolution, 4.0 mm slice thickness, echo-time shift = 1.0 ms. Utilising the known chemical shift between water and fat signal constituents, separate water and fat images were calculated within the scanner software. Subject height and body mass were measured in standing before the MRI scan using a stadiometer and calibrated weighing scales. Patients at our centre are routinely classified according to GMFCS level in their medical record by their consulting physician or surgeon.

### Data processing

**Figure 1:**
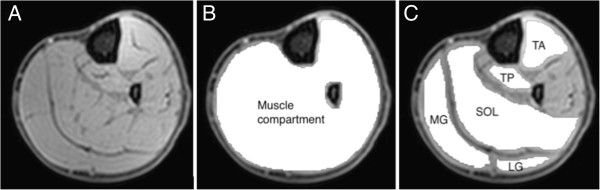
Image segmentation: (A) Example mDixon water image; (B) manually drawn region of interest (white) for the muscle compartment for IMAT segmentation; and (C) the 5 muscles analysed drawn around tibialis anterior (TA), tibialis posterior (TP), soleus (SOL), medial gastrocnemius (MG) and lateral gastrocnemius (LG) for IntraMF segmentation.

Regions of interest (ROIs) were manually drawn around the SF, muscle compartment (Figure 1B), and five individual muscles; the soleus, medial gastrocnemius, lateral gastrocnemius, tibialis anterior, and tibialis posterior (Figure 1C), on the mDixon water images using Osirix version 3.7.1 [25]. SF volume was normalised to muscle volume (Equation 1), creating a SF to muscle volume ratio (SF/M). The ROIs around the entire musculature and individual muscles were eroded with a structuring element size of 2 pixels (4.0 mm) to remove any potential edge effects with surrounding subcutaneous fat (or intermuscular fat for IntraMF quantification). IMAT and IntraMF were calculated using a ratio of the water and fat signal intensities within the eroded ROIs (Equation 2). The reproducibility of the Dixon technique employed was measured using 5 acquisitions of one TD subject imaged over 3 visits, with the scan repeated twice during 2 of these visits. Reproducibility was defined as the average standard deviation of the measured percentage fat, i.e. the standard deviation averaged across all muscles investigated.

(1) $$\mathrm{SF}/M=\frac{\text{SF}\;\text{Volume}}{\text{Muscle}\;\text{Volume}}$$

(2) $$\%\;\text{Fat}=100\times \left(\frac{I_{\text{Fat}}}{I_{\text{Fat}}+I_{\text{Water}}}\right)$$

Where I = Signal intensity.

### Data analysis

Following application of the Kolmogorov-Smirnov test for normality and Levene’s test for equality of variance, independent samples t-tests were performed to investigate physical characteristic differences between the two subject groups for all measured parameters. Two-way ANOVA was performed on IntraMF with GMFCS level and muscle identity as fixed factors with a Tukey post-hoc test. Non-parametric independent samples median tests compared SF/M and IMAT between groups. The Kruskal-Wallis Test was used to investigate differences in SF/M and IMAT with GMFCS level. Pearson’s correlations were used to investigate relationships between measured parameters. All statistical tests were performed using SPSS (Version 20.0; IBM SPSS) with significance set to p ≤ 0.05.

## Results

**Figure 2:**
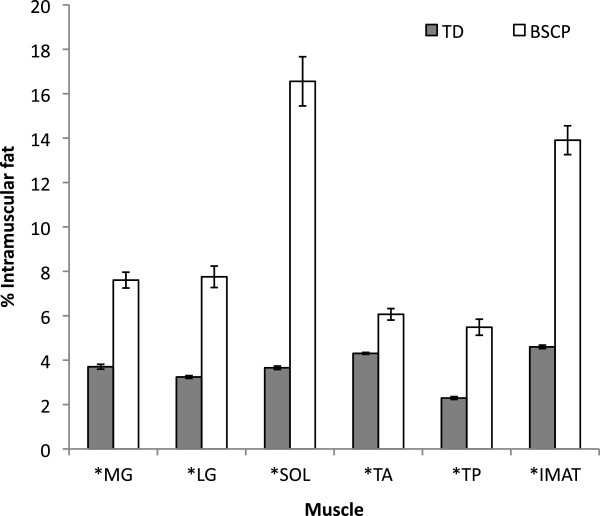
**Percentage IntraMF and IMAT in the medial gastrocnemius (MG), lateral gastrocnemius (LG), soleus (SOL), tibialis anterior (TA), tibialis posterior (TP) and in the BSCP group (white) and TD group (grey).** IMAT and IntraMF in all muscles were significantly different between groups (p < 0.05). Error bars represent the standard error of each group.

**Figure 3:**
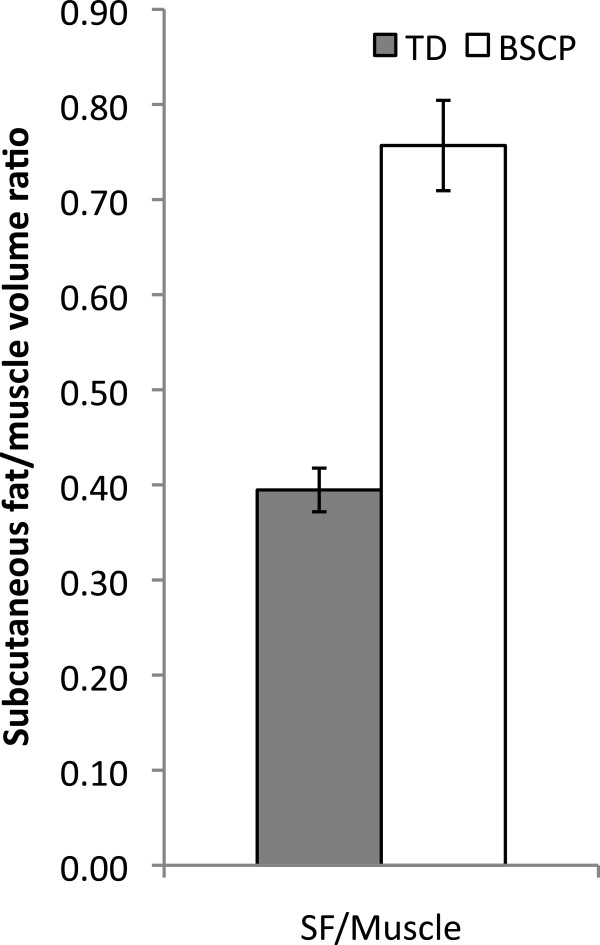
**Subcutaneous fat to muscle volume ratio in the BSCP group (white) and TD group (grey).** Error bars represent the standard error of each group.

**Figure 4:**
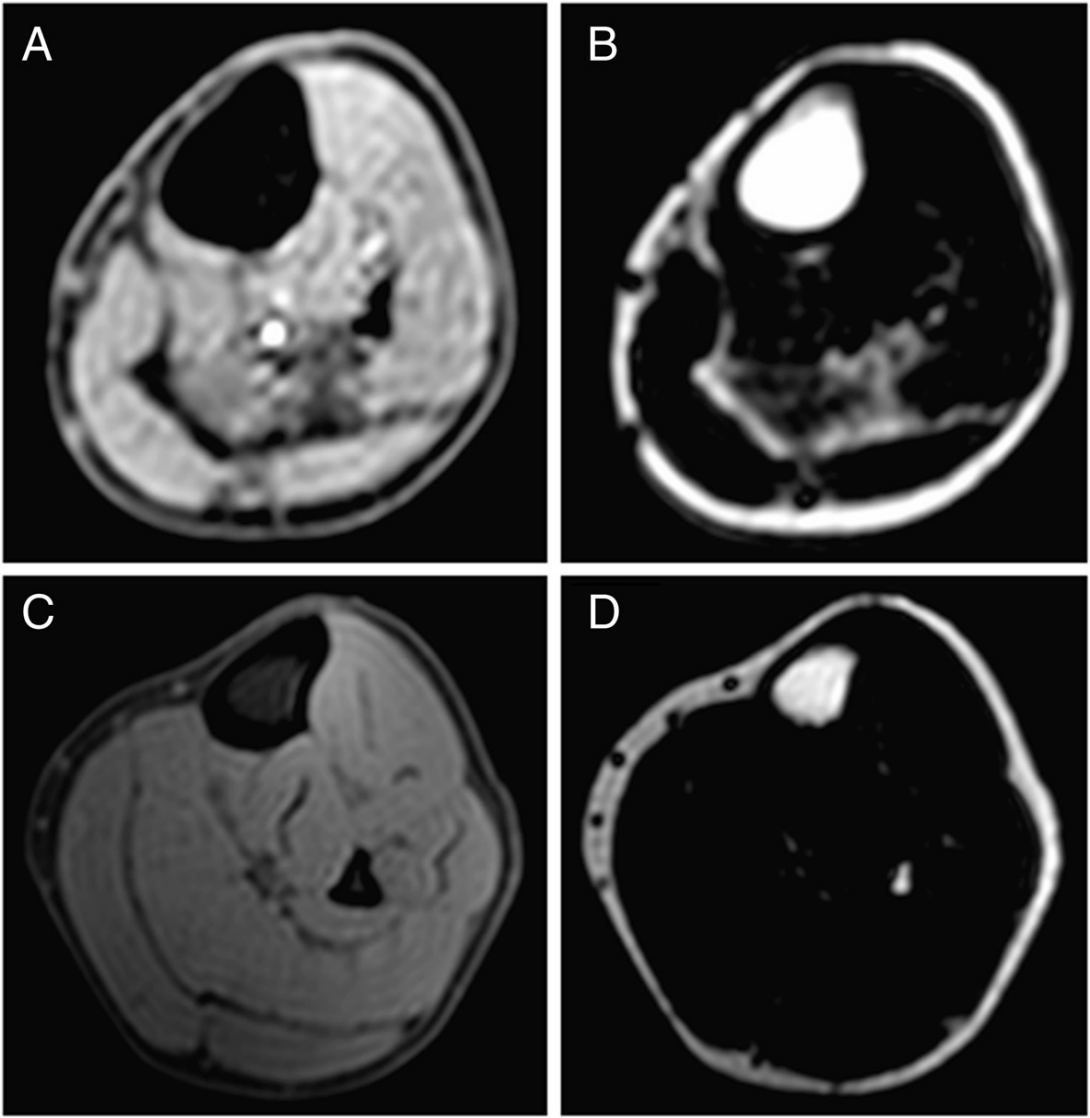
Example water (A and C) and fat (B and D) for one subject with BSCP (A and B), and an age matched TD subject (C and D).

Physical characteristics of subjects in the BSCP and TD groups are summarised in Table 2. There were no group differences in age, body mass, height, or BMI (p > 0.05). The GMFCS level of the BSCP subjects are also presented in Table 2. The average standard deviation representing reproducibility of intramuscular fat averaged across all muscles was 0.33%.Figure 2 shows a histogram of group-averaged percentage IntraMF and IMAT. The BSCP group had a significantly higher average percentage IMAT (p < 0.001) and IntraMF compared to the TD group for all muscles investigated (p < 0.001), with the soleus having the largest percentage fat difference (12.9% greater in BSCP group). In the BSCP group, IntraMF was significantly correlated between all muscles measured (r = 0.697 to 0.947, p < 0.001 to p = 0.025) except for the soleus, which did not correlate with any other muscle (r = 0.437 to 0.587, p = 0.074 to 0.206). Figure 3 shows a histogram of group-averaged SF/M ratio. The mean SF/M ratio was 1.9 times greater in the BSCP group compared to the TD group; however, this difference was not statistically significant (p = 0.179). Example images for a case and age-matched control are given in Figure 4.

**Table 2:** Physical characteristics (mean ± standard deviation) of BSCP and TD groups and number of BSCP subjects in each GMFCS levels (I-III)

	**BSCP group**	**TD group**
Number of subjects	10	10
Age (years)	22.5 ± 2.9	22.8 ± 3.0
Sex (m,f)	7, 3	6, 4
Body mass (kg)	64.0 ± 11.5	71.2 ± 11.8
Height (m)	1.69 ± 0.08	1.76 ± 0.12
BMI (kg/m^2^)	22.4 ± 3.6	22.9 ± 1.7
GMFCS level I	2	N/A
GMFCS level II	5	N/A
GMFCS level III	3	N/A

One-way ANOVA and post hoc analysis revealed significant differences in IntraMF levels by GMFCS level (p < 0.001), with GMFCS level III significantly higher compared to GMFCS levels I and II (p < 0.001 and p = 0.001 respectively). No significant difference was observed in IMAT or SF/M with GMFCS level (p = 0.131). In the TD group, SF/M was correlated with IMAT (r = 0.81, p = 0.005) but not with IntraMF (r = 0.182, p = 0.206), and no correlation was observed between IMAT and IntraMF (r = 0.251, p = 0.079). In the BSCP group, no correlations were observed between SF/M and IMAT (r = 0.392, p = 0.262) or IntraMF (r = 0.134, p = 0.353); IMAT and IntraMF were significantly correlated (r = 0.529, p < 0.001).

## Discussion

We conducted MRI measurements of IMAT, SF/M ratio, and IntraMF on 10 young adults with BSCP and 10 of their TD peers. This is the first study to show that ambulant adults with BSCP have raised levels of *IntraMF*, ranging from 2.3 to 34.4%. Percentage IntraMF was also found to be significantly different between GMFCS levels, with those at GMFCS III having greater fatty infiltration. These differences in IntraMF by GMFCS level suggest IntraMF maybe related to the degree of mobility impairment. This may be due to decreased physical activity with increasing mobility impairments.

The 1.9 fold greater SF/M ratio in the BSCP group is comparable to the results of Johnson *et al.*[18], who reported 2 fold greater SF/M ratio in the mid thigh of children with quadriplegic cerebral palsy GMFCS levels III-V [18]. This is surprising due to the higher functional ability of the subjects in this study (GMFCS levels I-III). IMAT values cannot be compared between these studies due to the separation by Johnson *et al.* of IMAT into intermuscular and sub-fascial fat and the different imaging and analysis techniques employed.

### Clinical implications

Children and young adults with cerebral palsy have been shown to have reduced muscle volumes in their lower limbs [2-4,26-30] and increased IMAT [18]. This study demonstrates that these muscles also have greater levels of intramuscular fat that is independent of subcutaneous fat levels. This combination of morphological and compositional changes may have implications both for the mechanical performance of these skeletal muscles and also for the predisposition of the adult with BSCP to cardio-metabolic disease [20].

Muscle weakness is a prevalent feature of individuals with cerebral palsy [26,31,32]. Muscle weakness, in this group, is in part caused by an inability to fully activate available muscular resources [33], increased co-activation [34] and by reduced muscle volume [2-4,26-30]. For a given muscle volume, a higher intramuscular fat content will correspond to reduced contractile tissue content, resulting in a weaker muscle than predicted from muscle mass alone. Furthermore, inflammatory cytokines produced by intramuscular fat may interfere with the action of myofibrillar proteins reducing specific force production [13]. These secondary pathologies may contribute to a deficit in the “functional reserve” of adults with BSCP, and may expose these individuals to a heightened risk of immobility with increasing age [30].

Greater intramuscular fat content in BSCP may expose these individuals to a greater risk of developing cardio-metabolic disease [20]. There is a strong relationship between intramuscular fat content, insulin resistance and type-II diabetes [15,35,36]. Within skeletal muscle, fat is stored in two separate compartments: Intramyocellular lipid (IMCL) and extramyocellular lipid (EMCL). EMCL is found within adipose cells adjacent to the muscle fibres, and IMCL is located along with enzymes involved in fatty acid esterification, hydrolysis, and transport into the mitochondria [37]. Enhanced storage of IMCL occurs due to the combined effects of high concentration of serum insulin and free fatty acids [38]. In particular, greater IMCL in the soleus correlates with glucose-insulin-lipid metabolism and insulin sensitivity [39-42], with soleus IMCL content being the only differentiating feature in a study of lean insulin-resistant subjects and their TD peers matched for BMI, body fat distribution, percentage body fat and physical fitness [42]. However, IntraMF quantification using Dixon imaging techniques measure IMCL and EMCL as a composite. IMCL has been shown to be unrelated to measures of adiposity [39,43]. In this study IntraMF did not correlate with SF/M. This suggests that the raised IntraMF measured in BSCP may be due to greater IMCL content. However, although the mDixon scans showed raised IntraMF with BSCP, whether this is due to raised IMCL, EMCL, or both is not yet known. Therefore, further investigations are required using magnetic resonance spectroscopy to determine the contributions of IMCL and EMCL to the raised IntraMF observed in this study.

Greater intramuscular fat may also be a marker of impaired mitochondrial content and/or function [44]. The increased intramuscular fat content observed in this study, particularly in the soleus, indicates that patients with cerebral palsy may have a greater risk of developing obesity related diseases, particularly type-II diabetes. Since IntraMF was observed to be greater with increasing GMFCS level, this risk of obesity-related disease may increase with decreasing functional ability. However, intramuscular fat is only one factor associated with the risk of developing obesity related diseases. Future studies of activity levels, fat levels, and glucose tolerance, are required to investigate the risk developing cardio-metabolic diseases in this patient group.

Greater intramuscular fat can be caused by a chronic mismatch between energy intake and expenditure [26,45]. Such a mismatch would also result in greater subcutaneous fat levels. In this study, however, despite SF/M being greater in the BSCP group, this was not statistically significant. This suggests that other factors as well as any potential energy mismatch are adversely affecting the intramuscular fat content in BSCP.

IntraMF has been correlated with deficits in central muscle activation [46], increased risk of future mobility loss [47,48], and insulin resistance [49] in the elderly population. Preliminary studies have also shown that particular exercise regimes in older adults may prevent and decrease intramuscular fat [10]. Since skeletal muscle in cerebral palsy has similarities with muscles in the elderly, including reduced muscle volume [50], increased stiffness [51] and reduced voluntary muscle activation [33], these exercise studies could have important implications for the physical management of this group as suggested by Peterson *et al.*[14].

### Limitations

The number of subjects recruited may limit the scope and power of this study. However, the differences in intramuscular fat between the TD and BSCP groups were large and statistically significant. The group differences were also much larger than the reproducibility of the mDixon technique used in this study, defined as the average standard deviation of measured percentage fat across all muscles (0.33%).

Despite having good accuracy and reproducibility, fat-fractions measured using multi-echo techniques are not standardised and platform-independent. The precision of the quantified fat fraction depends on five confounding factors; T1 bias, T2* decay, spectral complexity of fat, noise bias, and eddy currents [22]. The T1 bias and the assumption that fat has a single frequency peak, results in an inadvertent misidentification of some signal from fat as arising from water, and hence to quantification errors [52]. The T1 bias is due to the T1 weighting of the mDixon sequence, in order to reduce scan time. When there is partial-volume mixing of fat and muscle tissue within pixels, signal from fat, which has a shorter T1 than water, is overestimated. Therefore, despite being suitable for comparisons within single site studies, comparison of absolute values of intramuscular fat quantified in this study across sites where different techniques and sequence designs have been employed will be more difficult.

**Figure 5:**
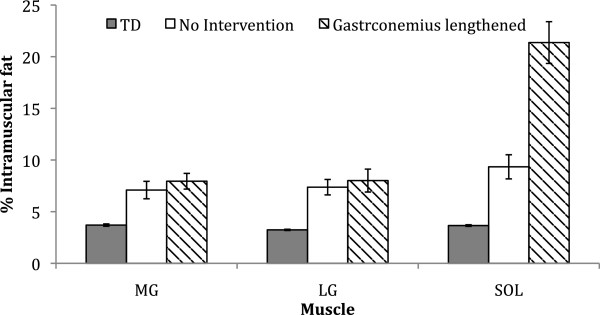
Percentage intramuscular fat in the medial gastrocnemius (MG), lateral gastrocnemius (LG) and soleus (SOL) for the TD group (grey), the no intervention BSCP subjects (white) and the gastrocnemius recession BSCP subjects (striped).

Six of the ten BSCP subjects had undertaken gastrocnemius recessions to the left leg; with one of these six subjects also having had Achilles tendon lengthening. Since muscle injury can lead to fatty degradation of muscle tissue [53-56], it is possible that the histories of surgical intervention received by the individuals with BSCP in this study may have influenced the development of particular muscles. A histogram comparing the percentage intramuscular fat between the TD group, BSCP subjects with no intervention, and BSCP subjects who have previously received gastrocnemius lengthening is given in Figure 5. Percentage intramuscular fat was significantly dependent on intervention and muscle (p = 0.048 and p = 0.009 respectively). Despite not being operated on directly, the soleus had significantly higher intramuscular fat content compared to MG and LG (p = 0.006 and p = 0.007 respectively). This data suggests that gastrocnemius lengthening may result in damage to the soleus causing fatty accumulation. Longitudinal studies are required to determine whether surgery results in greater accumulation of intramuscular fat, or whether those with heightened levels of intramuscular fat are more likely to have surgery.

The subjects groups were not age, BMI, and sex matched: however, the physical characteristic differences between the two groups were minimal with no significant difference in age, body mass, height, or BMI observed between the groups. It is possible that the large differences in fat levels in BSCP are due to large differences in life style, including activity levels and diet. Alternatively, the greater fat levels observed in this study may be due to an inherent predisposition with BSCP. To assess the contribution of the factors, further research is required to identify the cause of the increased intramuscular fat in BSCP.

## Conclusion

Greater intramuscular fat and IMAT is found in BSCP compared to their TD peers; with the amount of intramuscular fat related to GMFCS level. Despite normal BMIs, the patients in this group may have an increased risk of developing obesity-related diseases, with risk increasing with decreasing functional ability. Furthermore, the increasing intramuscular fat content may help to explain deficits in muscle performance in this group.

## Abbreviations

BMI: Body Mass Index; BSCP: Bilateral Spastic Cerebral Palsy; EMCL: Extramyocellular lipid; GMFCS: Gross Motor Function Classification System; IMCL: Intramyocellular lipid; LG: Lateral Gastrocnemius; MG: Medial Gastrocnemius; MRI: Magnetic Resonance Imaging; ROIs: Regions of interest; SOL: Soleus; SF: Subcutaneous fat; SF/M: Subcutaneous to muscle volume ratio; TA: Tibialis Anterior; TD: Typically developing; TP: Tibialis Posterior.

## Competing interests

The authors declare that they have no competing interests.

## Authors’ contributions

JJN was responsible for study design, MRI sequence development, data collection, data analysis and interpretation, and drafted the manuscript. GDE Assisted in study design, sequence development, data collection, and helped to draft the manuscript. SFK assisted in study design and helped to draft the manuscript. APL assisted in data analysis and helped to draft the manuscript. MG assisted in study design, subject recruitment, data interpretation, and helped to draft the manuscript. APS was responsible for conception and assisted in study design, data analysis and interpretation and helped to draft the manuscript. All authors read and approved the final manuscript.

## Pre-publication history

The pre-publication history for this paper can be accessed here:

http://www.biomedcentral.com/1471-2474/15/236/prepub
